# Assessment of obstructive sleep apnea-related sleep fragmentation utilizing deep learning-based sleep staging from photoplethysmography

**DOI:** 10.1093/sleep/zsab142

**Published:** 2021-06-05

**Authors:** Riku Huttunen, Timo Leppänen, Brett Duce, Arie Oksenberg, Sami Myllymaa, Juha Töyräs, Henri Korkalainen

**Affiliations:** 1 Department of Applied Physics, University of Eastern Finland, Kuopio, Finland; 2 Diagnostic Imaging Center, Kuopio University Hospital, Kuopio, Finland; 3 School of Information Technology and Electrical Engineering, The University of Queensland, Brisbane, Australia; 4 Department of Respiratory & Sleep Medicine, Sleep Disorders Centre, Princess Alexandra Hospital, Brisbane, Australia; 5 Institute for Health and Biomedical Innovation, Queensland University of Technology, Brisbane, Australia; 6 Sleep Disorders Unit, Loewenstein Hospital – Rehabilitation Center, Raanana, Israel; 7 Science Service Center, Kuopio University Hospital, Kuopio, Finland

**Keywords:** obstructive sleep apnea, sleep fragmentation, sleep staging, deep learning, survival analysis

## Abstract

**Study Objectives:**

To assess the relationship between obstructive sleep apnea (OSA) severity and sleep fragmentation, accurate differentiation between sleep and wakefulness is needed. Sleep staging is usually performed manually using electroencephalography (EEG). This is time-consuming due to complexity of EEG setup and the amount of work in manual scoring. In this study, we aimed to develop an automated deep learning-based solution to assess OSA-related sleep fragmentation based on photoplethysmography (PPG) signal.

**Methods:**

A combination of convolutional and recurrent neural networks was used for PPG-based sleep staging. The models were trained using two large clinical datasets from Israel (*n* = 2149) and Australia (*n* = 877) and tested separately on three-class (wake/NREM/REM), four-class (wake/N1 + N2/N3/REM), and five-class (wake/N1/N2/N3/REM) classification. The relationship between OSA severity categories and sleep fragmentation was assessed using survival analysis of mean continuous sleep. Overlapping PPG epochs were applied to artificially obtain denser hypnograms for better identification of fragmented sleep.

**Results:**

Automatic PPG-based sleep staging achieved an accuracy of 83.3% on three-class, 74.1% on four-class, and 68.7% on five-class models. The hazard ratios for decreased mean continuous sleep compared to the non-OSA group obtained with Cox proportional hazards models with 5-s epoch-to-epoch intervals were 1.70, 3.30, and 8.11 for mild, moderate, and severe OSA, respectively. With EEG-based hypnograms scored manually with conventional 30-s epoch-to-epoch intervals, the corresponding hazard ratios were 1.18, 1.78, and 2.90.

**Conclusions:**

PPG-based automatic sleep staging can be used to differentiate between OSA severity categories based on sleep continuity. The differences between the OSA severity categories become more apparent when a shorter epoch-to-epoch interval is used.

**Statement of Significance**

Differentiation between sleep and wakefulness, which is needed to assess obstructive sleep apnea (OSA)-related sleep fragmentation, is commonly performed using EEG signal segmented to 30-s epochs. With this protocol, some of the sleep stage transitions may be omitted. As home measurements are becoming increasingly common, assessment of sleep fragmentation with a simple measurement setup is needed. In this study, automatic PPG-based sleep staging was used to assess sleep fragmentation in a clinical population with suspected OSA. Overlapping epochs were used with the automatic deep learning models to obtain a higher resolution of the sleep architecture. The results show that PPG-based automatic sleep staging is possible and can be utilized to differentiate between OSA severity categories with respect to sleep fragmentation.

## Introduction

Obstructive sleep apnea (OSA) is a sleep disorder characterized by recurrent complete or partial breathing obstructions which heavily affect the sleep architecture [1]. Over 900 million people worldwide are estimated to suffer from OSA [2]. One consequence of OSA is sleep fragmentation due to the arousals induced by the breathing obstructions. Sleep fragmentation is associated with various OSA-related symptoms, including daytime sleepiness and decreased psychomotor vigilance [1]. One proposed method to assess the relationship between OSA and fragmented sleep is to perform survival analysis on the duration of continuous sleep of subjects grouped by OSA severity category [3].

In clinical practice, sleep stages are usually scored manually by visual inspection using the signals recorded during a polysomnography (PSG), including the electroencephalogram (EEG), the electrooculogram (EOG), and the electromyogram (EMG) [4]. Manual scoring of sleep stages is a time-consuming task performed by trained professionals. However, even with years of experience on the task, two scorers are prone to score some of the sleep stages differently [5–8]. The interrater reliability is especially low for N1, with reported agreement as low as 63% [8]. Cohen’s κ value is a widely used metric for interrater agreement [9]. In one study, PSGs of 72 subjects (56 healthy controls, 16 patients with different sleep disorders) from three different hospitals were scored according to the American Academy of Sleep Medicine (AASM) 2007 rules by two independent scorers. A total of seven scorers participated in the study. Overall agreement for five-stage scoring was κ = 0.76, greatly varying between different sleep stages from moderate agreement (κ = 0.46) on N1 sleep to almost perfect agreement (κ = 0.91) on rapid eye movement (REM) sleep [5].

Since manual sleep scoring is laborious, automated solutions for sleep staging have been developed by utilizing a myriad of different approaches [10]. Combinations of nonlinear features such as wavelet transforms, entropy, spectral features, and autoregression coefficients have been used with classifiers such as k-nearest neighbors and random forests [11–15]. In classification to five stages (wake/N1/N2/N3/REM), the reported accuracies of these methods, which involve handcrafted feature engineering and primarily use EEG, have varied from 75% to 83%. Other examples of methods used for automated sleep staging include classification of cardiac features calculated from an ECG recording [16, 17], and classification of features derived from bed sensors measuring heart rate and movements [18]. More recently, deep learning-based methods have been utilized for automated EEG-based sleep staging with very good results with reported accuracies varying from 84% to 87% and Cohen’s κ values between manual and automated sleep staging varying from 0.77 to 0.82 [19–22].

While the characterization of sleep stages is often focused on the brain, sleep also affects the autonomous nervous system (ANS) activity [23, 24]. The sympathetic nervous system (SNS) activity is decreased in non-REM (NREM) sleep, and there are phasic bursts of SNS activity in REM sleep [24]. Due to the changes in ANS activity, there are also hemodynamic changes during sleep [24, 25]. During NREM sleep, mean arterial pressure and cardiac output are reduced. In contrast, during REM sleep the arterial pressure and heart rate are increased [25]. These changes are reflected in the photoplethysmogram (PPG) which measures the changes in blood volume in the microvascular tissue [26]. Thus, PPG can be used to differentiate between wake, NREM sleep, and REM sleep [19].

PPG measurements are simple to set up compared to standard EEG measurements, which makes PPG-based automated sleep staging an interesting alternative to EEG-based staging. A few studies have used PPG for three-stage classification three-stage classification (wake/NREM/REM) using handcrafted features [27, 28]. These methods have achieved accuracies varying from 73% and 75%, and Cohen’s κ values varying from 0.53 to 0.55. Recently, our research group introduced a sleep staging approach utilizing deep learning with raw PPG as the input. This method achieved an accuracy of 80.1% and Cohen’s κ of 0.65 in three-stage classification [19]. However, the wakefulness classification accuracy of this method was 72%, and increasing this accuracy would be highly beneficial for the assessment of sleep fragmentation.

In the present work, we aimed to assess OSA-related sleep fragmentation with survival analysis of sleep continuity estimated using the PPG signal. The first hypothesis was that automatic PPG-based sleep staging can capture the interruptions of sleep at a level of accuracy that allows differentiation between OSA severity groups in terms of sleep continuity. Since the classification of sleep and wake is crucial for the task, an auxiliary objective was to improve the accuracy of previous PPG-based sleep staging methods. The second hypothesis was that hypnograms with higher resolution would better highlight the differences between OSA severity groups based on sleep continuity by capturing the short interruptions of sleep that may be omitted from sleep staging when using traditional 30-s epochs. This was tested by overlapping the PPG epochs during prediction to artificially obtain shorter epoch-to-epoch intervals. Recently, this method has been used to analyze sleep fragmentation with automatic EEG-based sleep staging [29]. The survival analyses were performed separately for hypnograms generated with different amounts of overlap.

## Methods

### Data

In the present study, two separate clinical datasets, denoted as datasets A and B, were utilized. Dataset A, which was used only to pre-train the sleep staging model, consisted of 2149 full PSG recordings of suspected OSA patients consecutively collected during the years 2001–2011 at the Sleep Disorders Unit, Loewenstein Hospital – Rehabilitation Center (Raanana, Israel). The PSGs were recorded with Rembrandt Manager System (Medcare, Amsterdam, Netherlands), and the sleep stages were scored according to the Rechtschaffen & Kales rules [30]. To conform with the more recent rules by the AASM [31], NREM stages 3 and 4 were combined into a single N3 stage before training the sleep staging models. The Ethical Committee of Loewenstein Hospital approved the use of dataset A (No. 0006-17-LOE).

Dataset B consisted of 933 consecutive PSG recordings of suspected OSA patients. The recordings were collected in 2012–2018 at the Sleep Disorders Centre, Princess Alexandra Hospital (Brisbane, Australia). Compumedics Grael devices with Profusion 4.1 software (Compumedics, Abbotsford, Australia) were used to record and analyze the signals. All recordings were manually scored in accordance with the prevalent AASM rules [31]. A total of 877 recordings were included in the study after leaving out recordings that included corrupted signals or contained less than 1 h of sleep. The Institutional Human Research Ethics Committee at Princess Alexandra Hospital approved the use of dataset B (HREC/16/QPAH/021 and LNR/2019/QMS/54313).

Since the dataset B was acquired with more recent hardware, and analyzed using the more recent AASM guidelines, it was the main dataset used in the present study, and the only one used in validation and testing. The demographic information (Table 1) and results are only reported for the dataset B.

**Table 1.** Demographic information on the studied population of patients with suspected OSA (dataset B)

	Median (inter-quartile range)			
	Whole population (*n* = 877)	Training set (*n* = 632)	Validation set (*n* = 70)	Test set (*n* = 175)
Age (years)	55.9 (44.7–65.7)	55.8 (44.6–65.6)	56.2 (45.6–65.8)	55.8 (44.8–65.9)
BMI (kg/m^2^)	34.4 (29.3–40.4)	34.7 (29.4–40.6)	31.4 (27.0–35.7)	33.8 (29.7–39.8)
TST (min)	309.0 (254.5–359.5)	309.0 (255.8–358.9)	308.2 (258.0–373.0)	309.5 (252.2–355.5)
SE (%)	70.7 (58.4–82.0)	71.0 (58.4–82.0)	72.5 (60.7–85.5)	69.7 (57.8–80.8)
WASO (min)	102.5 (61.0–149.5)	103.2 (61.0–148.1)	86.0 (46.0–145.5)	105.5 (67.5–158.8)
ArI (1/h)	20.6 (13.9–31.3)	20.5 (13.8–31.3)	18.4 (13.5–27.0)	22.2 (15.1–36.3)
N1 sleep (%)	10.9 (6.8–18.7)	10.9 (6.8–18.6)	9.6 (7.1–15.3)	12.1 (6.3–25.8)
N2 sleep (%)	48.3 (41.4–56.1)	48.2 (41.6–55.9)	52.0 (42.1–59.0)	47.9 (40.6–55.3)
N3 sleep (%)	18.3 (9.7–27.0)	18.6 (9.9–27.3)	17.5 (8.9–27.3)	17.4 (9.6–25.9)
REM sleep (%)	17.2 (12.0–22.1)	17.4 (12.4–22.0)	17.1 (11.4–22.1)	16.5 (11.4–22.4)
NREM sleep (%)	82.8 (77.8–87.9)	82.6 (78.0–87.6)	82.9 (77.9–88.6)	83.4 (77.6–88.6)
TRT (min)	442.5 (410.5–474.5)	443.2 (413.0–475.2)	430.0 (399.9–463.2)	443.0 (410.2–474.8)
AHI (1/h)	15.7 (7.0–32.6)	15.4 (7.0–32.2)	11.0 (5.2–23.3)	19.9 (8.2–41.2)
	**Count (%)**			
	**Whole population**	**Training set**	**Validation set**	**Test set**
Male	480 (54.7)	347 (54.9)	38 (54.3)	95 (54.3)
Female	396 (45.2)	285 (45.1)	31 (44.3)	80 (45.7)
No OSA	148 (16.9)	103 (16.3)	16 (22.9)	29 (16.6)
Mild OSA	275 (31.4)	207 (32.8)	26 (37.1)	42 (24.0)
Moderate OSA	205 (23.4)	146 (23.1)	16 (22.9)	43 (24.6)
Severe OSA	249 (28.4)	176 (27.8)	12 (17.1)	61 (34.9)

BMI = body mass index, TST = total sleep time, SE = sleep efficiency, WASO = wake after sleep onset, ArI = arousal index, REM = rapid eye movement, NREM = non-rapid eye movement, TRT = total recording time, AHI = apnea-hypopnea index, OSA = obstructive sleep apnea.

no OSA: AHI < 5, mild OSA: 5 ≤ AHI < 15, moderate OSA: 15 ≤ AHI < 30, severe OSA: AHI ≥ 30. no OSA: AHI < 5, mild OSA: 5 ≤ AHI < 15, moderate OSA: 15 ≤ AHI < 30, severe OSA: AHI ≥ 30.

The patients were assigned to separate training, validation, and test sets before training the sleep staging models and further analyses. A random sample of 20% of the patients from dataset the B was used as the independent test set. After the test set selection, 10% of the remaining data were sampled as the validation set. Details on the training, validation, and test set distributions are presented in Table 1. During training, only the training set was used to adjust the model weights. The validation set was used to monitor the training process and choose the final model. The test set was used for performance assessment of the final model and in the subsequent analysis of sleep fragmentation.

The data underlying this article cannot be shared publicly due to privacy reasons.

### Sleep staging

Automatic sleep staging models were trained using only the PPG signal as the input. The raw signals were exported from the PSG software with their original sampling rate of 256 Hz. No additional filtering was performed during the exports. The PPG signal was downsampled from 256 to 32 Hz, after applying an order 8 Chebyshev type I antialiasing filter. Then, *z*-score normalization was applied to the downsampled signals. No further preprocessing was performed. Sleep was separately classified into three classes (wake/NREM/REM), four classes (wake/N1 + N2/N3/REM), and five classes (wake/N1/N2/N3/REM). All models were pre-trained using the dataset A, and the best-performing models according to cross-entropy loss on the validation set were used to initialize the weights before fine-tuning using the dataset B. Smaller learning rates were used when fine-tuning the pre-trained model with dataset B to avoid destroying the feature representation learned from dataset A.

A general architecture consisting of a convolutional neural network (CNN), a recurrent neural network (RNN), and a densely connected classifier was used [19, 22]. The model was implemented in Python using Tensorflow 2.3.0 and its Keras API. The deep learning model architecture is described in Table 2. The CNN extracted features from 30-s windows of the raw PPG signal. This representation was aligned with the 30-s epochs used in manual sleep staging. The CNN was based on EfficientNet [32], which is a state-of-the-art deep learning architecture for image classification. In the present work, the 2D EfficientNet architecture was modified for 1D inputs by substituting the 2D convolutions with 1D convolutions. The Swish activation function [33] was used similarly to the original EfficientNet. The output features of the CNN were used as the input for the RNN. A bidirectional RNN was used to capture the sleep state dynamics both backward and forward in time. Long short-term memory (LSTM) cells were chosen over gated recurrent units (GRU) after evaluating both. The bidirectional LSTM output features were then fed to two densely connected layers with rectifier linear unit (ReLU) activations. The classifier output was produced by applying the softmax activation function to the final dense layer’s output.

**Table 2.** The architecture of the sleep staging model. A time-distributed layer wrapper applies the layer defined in parentheses to each sleep staging epoch separately. The term ‘Any’ in the output shape signifies a variable-length sequence. In addition to the high-level architecture, the detailed architecture of the CNN is provided. The kernel size, strides, number of output channels, expand ratio, squeeze-and-excitation (S&E) ratio, and the number of repeats for each block are hyperparameters of the EfficientNet [32] model

High-level architecture							
	Layer type		Output shape		#params		
PPG	Input		(Any, 960, 1)		0		
CNN	TimeDistributed (CNN)		(Any, 512)		696,156		
RNN	Bidirectional (LSTM)		(Any, 256)		656,384		
Dense	TimeDistributed (Dense)		(Any, 32)		8,224		
Hypnogram	TimeDistributed (Dense)		(Any, 5)		165		
Detailed architecture of the CNN							
	**Resolution**	**Kernel size**	**Strides**	**Channels out**	**Expand ratio**	**S&E ratio**	**Repeats**
Stem Conv	960	11	3	32	N/a	N/a	1
MBConv1	317	3	1	16	1	0.25	1
MBConv2	317	5	2	24	6	0.25	2
MBConv3	159	3	2	24	6	0.25	2
MBConv4	80	5	2	32	6	0.25	3
MBConv5	40	5	2	48	6	0.25	4
MBConv6	20	5	2	64	6	0.25	5
MBConv7	10	3	1	128	6	0.25	1
Conv + GlobalAvgPool	10	1	1	512	N/a	N/a	1

PPG = photoplethysmogram, CNN = convolutional neural network, RNN = recurrent neural network, LSTM = long short-term memory, Conv = convolution, MBConv = inverted residual with bottleneck, GlobalAvgPool = global average pooling, N/a = not applicable. The second dimension of the resolution in the CNN is always one, which has been omitted for readability.

Hyperparameter tuning was performed using a disciplined approach [34]. First, a suitable range for learning rates was searched using a learning rate range test [35]. The resulting range was used with a one-cycle learning rate scheduling policy, in which the learning rate was initially set to the minimum of the range. Then, the learning rate was increased linearly after each network training epoch until the maximum of the range was reached. After that, the learning rate was linearly decreased back to the minimum using the same number of training epochs that was used when increasing the learning rate. Finally, the learning rate was exponentially decreased for 20 training epochs until it was two orders of magnitude smaller than the minimum learning rate indicated by the learning rate range test [34].

### Sleep parameters

Total sleep time (TST), sleep efficiency (SE), and wake after sleep onset (WASO) were computed for each patient in the test set using the five-stage model. In addition, the percentage of wake from the total recording time, as well as the percentages of each sleep stage from the TST were computed. The parameters were computed separately for each OSA severity category. OSA severity was defined using the apnea-hypopnea index (AHI; no OSA: AHI < 5, mild OSA: 5 ≤ AHI < 15, moderate OSA: 15 ≤ AHI < 30, severe OSA: AHI ≥ 30). The AHI values were calculated from the manually scored PSGs. Thirty-second non-overlapping epochs were used both in the manual scoring and when training the automatic sleep staging models. In addition, the automated model was used to produce hypnograms with higher temporal resolution by applying 15-, 5-, and 1-s epoch-to-epoch intervals by overlapping the consecutive 30-s epochs by 15, 25, and 29 s, respectively. The same model was used with all epoch-to-epoch intervals without retraining.

### Survival analysis of sleep continuity

Sleep continuity was evaluated using survival analysis techniques introduced by Norman et al. [3]. The mean length of continuous sleep was calculated for each patient. Then, Cox proportional hazards models were fitted with the mean continuous sleep as the time to event, and the one-hot encoded OSA severity categories as the binary covariates. The non-OSA group was used as the reference. The five-stage model was used in the survival analysis. The Cox proportional hazards modeling was performed separately with both manually scored hypnograms and the PPG-based hypnograms having 30-, 15-, and 5-s epoch-to-epoch intervals. In addition to acquiring the hazard ratios for decreased mean continuous sleep using the proportional hazards model, sleep continuity was evaluated visually from Kaplan–Meier plots.

### Statistical analysis

All statistical analyses were performed using Python 3.8.5. Overall accuracy, precision, recall, and F1 scores were used for performance assessment of the sleep staging models. In addition, Cohen’s κ was used to estimate the agreement between the manually scored PSG-based and the automatically scored PPG-based sleep staging. Medians and interquartile ranges were computed for the sleep parameters. Mean absolute error (MAE) was computed to assess the difference between the manual PSG-based and automated PPG-based sleep parameters. The Wilcoxon signed-rank test was used to test the statistical significance of differences in the sleep parameters between manual and automatic sleep staging. The Mann–Whitney *U* test was used to test the statistical significance of differences in the sleep parameters between the OSA severity categories. SciPy 1.4.1 [36] was used for the statistical tests. Non-parametrical tests were used since the target variables did not follow any parametric distribution, which was concluded after visual inspection and normality tests of the distributions (Shapiro–Wilk test). For the survival analyses (Cox proportional hazards modeling and Kaplan-Meier plots), lifelines 0.25.4 [37] was used.

## Results

### Sleep staging

**Figure 1.**
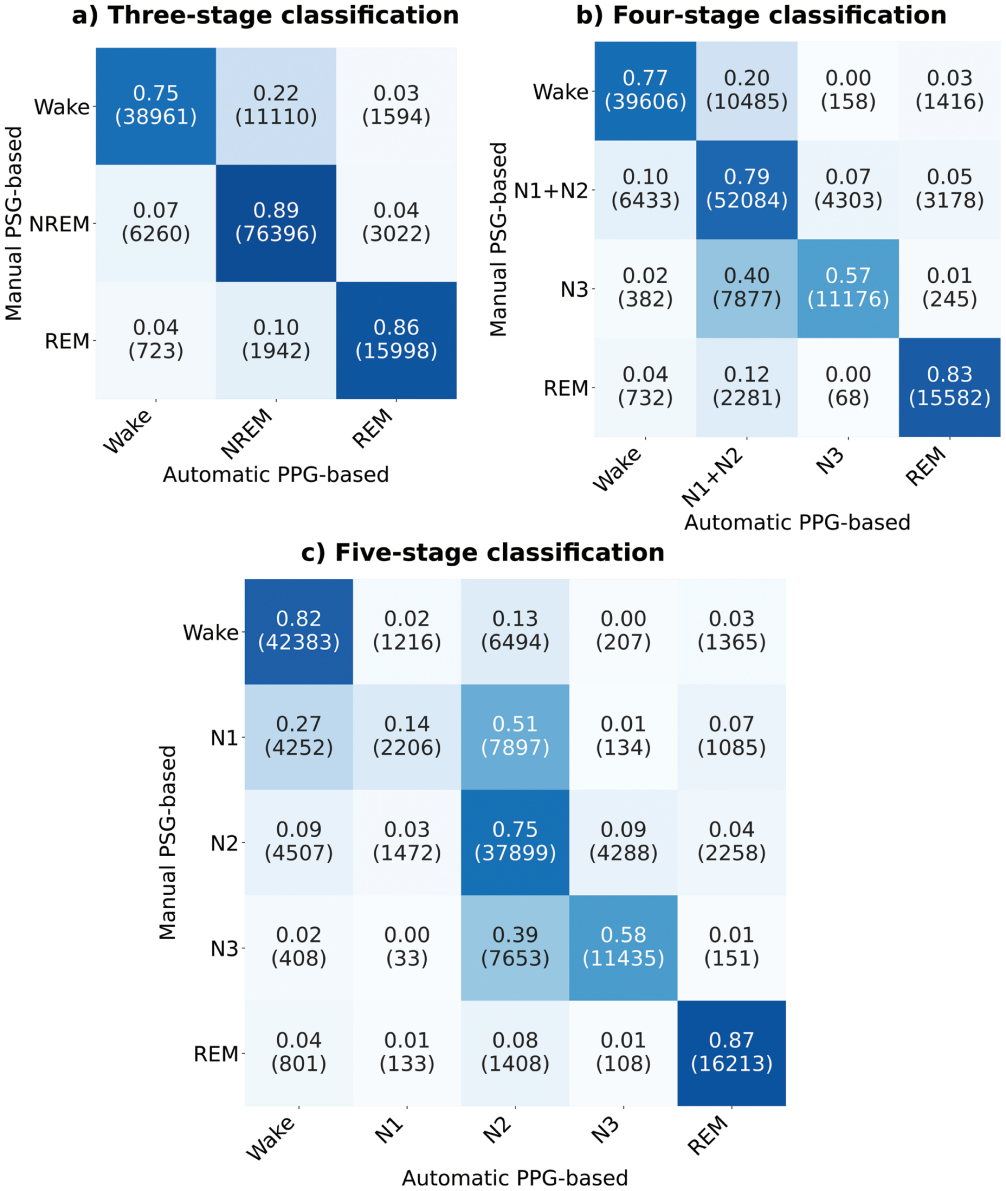
Confusion matrices for (a) three-class (wake/NREM/REM), (b) four-class (wake/N1+N2/N3/REM), and (c) five-class (wake/N1/N2/N3/REM) classification between PSG-based manual scoring and PPG-based automatic scoring on the test set (*n* = 175). Results are shown as fractions (epoch counts).

The confusion matrices for three-, four-, and five-class sleep stage classification are shown in Figure 1. In the three-stage classification, the overall accuracy of the classifier was 86.5% on the training set, 85.4% on the validation set, and 83.3% on the test set. Precision (recall) values for each class on the test set were 0.85 (0.75) for wake, 0.85 (0.89) for NREM, and 0.78 (0.86) for REM sleep. F1 scores for each class were 0.80 for wake, 0.87 for NREM, and 0.82 for REM. Cohen’s κ coefficient in the three-stage classification on the test set was κ = 0.72.

In the four-stage classification, the overall accuracy of the classifier was 77.8% on the training set, 73.5% on the validation set, and 74.1% on the test set. Class-wise test set precision (recall) values were 0.84 (0.77) for wake, 0.72 (0.79) for light sleep (i.e. N1+N2), 0.71 (0.57) for N3, and 0.76 (0.83) for REM sleep. Class-wise F1 scores were 0.80 for wake, 0.75 for light sleep, 0.63 for N3, and 0.80 for REM sleep. Cohen’s kappa coefficient in the four-stage classification for the test set was *κ* = 0.64.

In the five-stage classification, the overall accuracy of the classifier was 74.8% on the training set, 69.6% on the validation set, and 68.7% on the test set. Precision (recall) values for each sleep stage in the test set were 0.81 (0.82) for wake, 0.44 (0.14) for N1, 0.62 (0.75) for N2, 0.71 (0.58) for N3, and 0.77 (0.87) for REM. Class-wise F1 scores were 0.81 for wake, 0.22 for N1, 0.68 for N2, 0.64 for N3, and 0.82 for REM. Cohen’s kappa coefficient for the test set was *κ* = 0.60.

**Figure 2.**
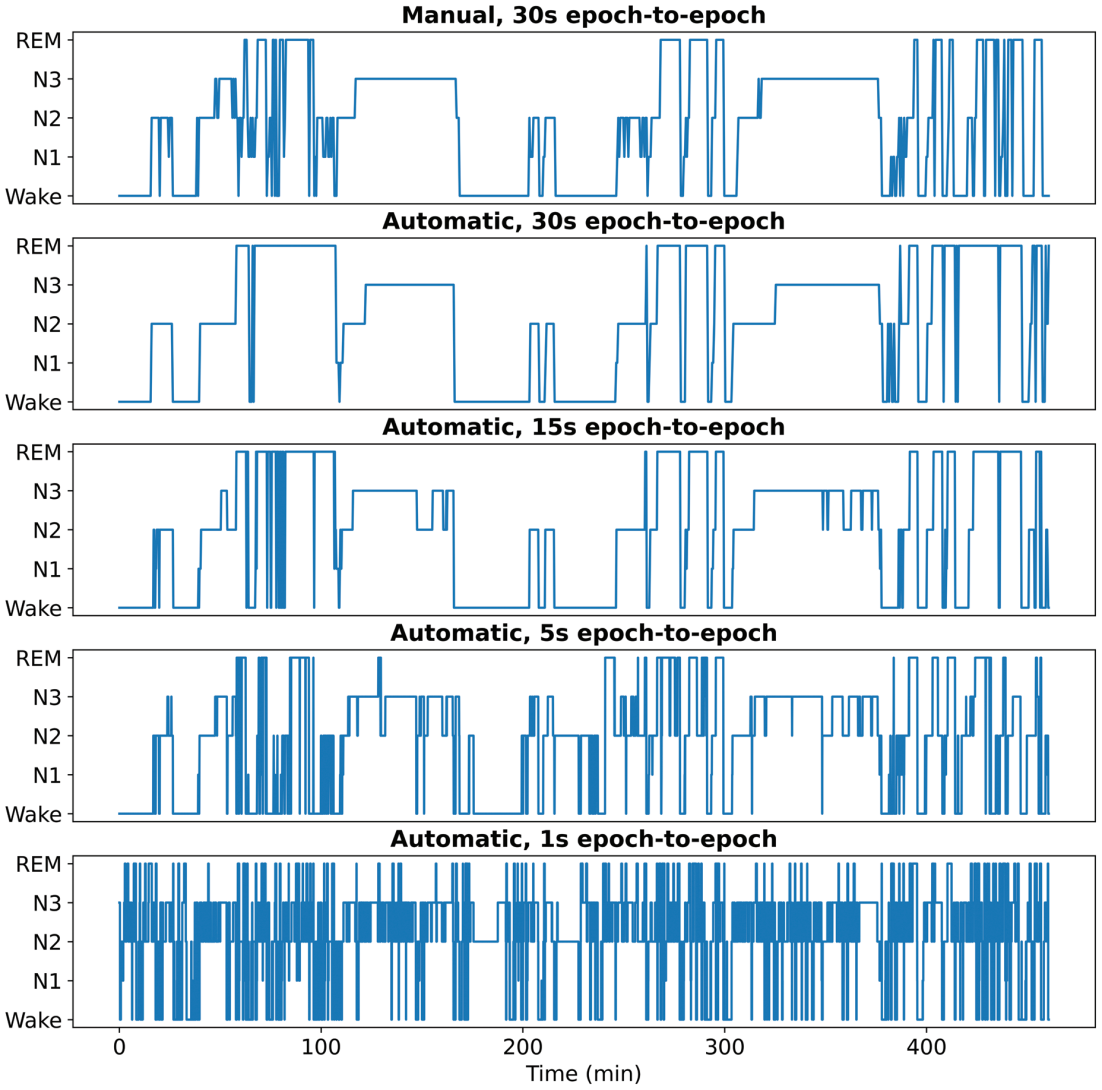
Example hypnograms (non-OSA patient) for PSG-based manual scoring and PPG-based automatic sleep staging with epoch-to-epoch intervals of 30, 15, 5, and 1 s. Using 1-s interval leads to severe artifacts of oscillations between sleep and wake.

A representative example of manual PSG-based and automatic PPG-based hypnograms for one healthy patient from the test set in the five-stage case are shown in Figure 2. The hypnogram produced with the automatic model using the original 30-s epoch-to-epoch interval had a smoothing effect on the sleep stage transitions compared to the manually scored hypnogram. When the same model was used with the 30-s epochs with 15-, 5-, and 1-s epoch-to-epoch intervals, the model produced increasingly more sleep-wake transitions. With the 1-s epoch-to-epoch interval, the original manual PSG-based sleep architecture became unrecognizable, so the 1-s interval was left out of further analyses after visual inspection of the hypnograms of a randomly chosen subsample of the patients. Since the number of epochs was increased proportional to the overlap used between consecutive epoch, the inference time of the model was also increased. With the test set of 175 patients, the inference took around 1 min with the original 30-s epoch-to-epoch interval, and around 6 min with the 5-s epoch-to-epoch interval.

Median and interquartile ranges for wake and different sleep stage percentages in each of the OSA severity categories computed from the manual PSG-based and automatic PPG-based hypnograms are shown in Table 3. The proportions of wake and REM sleep were estimated most consistently across different OSA category and epoch-to-epoch interval combinations. The proportion of N1 sleep was estimated the worst, especially in the severe OSA group. This was compensated by the automatic model by overestimating the proportion of N2 sleep. When the epoch-to-epoch interval was decreased to 5 s with the automatic hypnograms, the mean absolute errors between the manual and automatic sleep stage percentages were higher, except for N2 percentage.

**Table 3.** Median (inter-quartile range) for percentages of wake from total recording time (TRT), and of different sleep stages from total sleep time (TST), in manually and automatically scored hypnograms. The results are calculated for the test set (*n* = 175) using the five-stage model with 30-s epoch-to-epoch interval. Mean absolute error (MAE) compared to manual scoring is reported for the automatic models

		Wake, % of TRT		N1, % of TST		N2, % of TST		N3, % of TST		REM, % of TST	
		Median (IQR)	MAE	Median (IQR)	MAE	Median (IQR)	MAE	Median (IQR)	MAE	Median (IQR)	MAE
No OSA (*n* = 29)	**Manual**	26.5 (17.3–35.2)	–	5.8 (3.2–9.6)	–	53.4 (44.0–59.5)	–	19.6 (12.6–27.0)	–	17.7 (11.9–25.3)	–
	**30 s auto**	28.0 (21.6–38.4)	5.6	1.0 (0.5–2.0)*	6.1	57.0 (48.9–65.2)*	12.8	20.4 (13.2–25.7)	12.3	23.0 (13.3–28.5)*	4.0
	**15 s auto**	28.5 (21.0–39.6)	6.8	0.6 (0.4–1.2)*	6.6	54.3 (44.7–61.2)	12.9	26.3 (19.5–31.7)	12.3	20.9 (11.7–26.7)	4.3
	**5 s auto**	23.7 (18.7–30.9)	8.5	0.5 (0.3–0.6)*	6.9	44.8 (39.9–53.2)*	12.8	40.4 (32.1–45.2)*	20.6	11.2 (8.6–18.9)*	6.1
Mild OSA (*n* = 42)	**Manual**	22.4 (15.6–37.7)	–	9.1 (6.4–12.5)†	–	50.4 (44.8–53.5)	–	22.5 (15.5–29.0)	–	19.3 (13.9–22.6)	–
	**30 s auto**	27.2 (18.6–35.2)*	6.0	1.9 (0.9–3.2)*†	7.8	57.2 (54.0–62.0)*	11.1	19.0 (11.5–22.6)*	8.1	22.0 (17.7–25.0)*	3.9
	**15 s auto**	29.5 (22.2–38.2)*	6.6	1.4 (0.8–2.0)*†	8.3	51.9 (46.9–59.7)	9.9	25.0 (17.8–29.9)	9.2	21.2 (16.5–24.5)*	4.2
	**5 s auto**	26.8 (23.1–32.7)	7.7	0.9 (0.5–1.2)*†	9.1	49.5 (42.2–53.7)	9.4	34.5 (30.9–42.1)*	15.7	15.1 (11.7–17.9)*	5.1
Moderate OSA (*n* = 43)	**Manual**	34.1 (23.5–41.8)	–	9.3 (5.4–16.3)†	–	47.5 (42.0–56.0)	–	17.3 (10.8–26.3)	–	18.3 (11.6–26.3)	–
	**30 s auto**	34.0 (23.9–44.2)	6.0	4.0 (1.7–5.3)*†	9.2	55.5 (46.9–64.2)*	11.9	18.5 (10.5–26.0)	8.3	18.6 (14.9–28.9)*	5.6
	**15 s auto**	35.5 (29.7–47.4)*†	7.5	2.5 (1.7–4.0)*†	9.9	51.8 (41.7–59.9)	10.2	24.3 (15.6–31.8)*	9.8	19.2 (14.2–26.3)	4.9
	**5 s auto**	33.6 (26.8–41.1)†	8.5	1.5 (1.1–2.0)*†	11.2	51.0 (46.2–59.0)†	9.2	35.0 (25.3–40.6)*†	14.6	13.2 (9.7–16.1)*	7.1
Severe OSA (*n* = 61)	**Manual**	34.0 (23.9–51.8)†	–	28.3 (16.0–35.1)†	–	43.0 (36.3–52.3)†	–	11.8 (3.9–22.6)†	–	12.9 (9.7–19.9)	–
	**30 s auto**	32.9 (25.8–46.5)	8.2	8.5 (4.9–13.5)*†	17.8	63.0 (56.4–70.4)*†	20.3	7.9 (4.2–13.5)*†	9.2	15.7 (11.2–24.0)*	4.5
	**15 s auto**	37.0 (31.0–54.4)†	7.8	6.8 (3.8–11.7)*†	19.3	63.1 (54.4–68.6)*†	18.9	12.0 (7.9–16.5)†	8.8	16.1 (11.3–22.3)*	4.5
	**5 s auto**	34.5 (29.4–44.8)†	11.4	3.2 (1.7–4.9)*†	23.6	64.1 (55.7–69.4)*†	19.1	20.4 (15.5–29.1)*†	11.5	11.5 (8.4–13.8)*	5.2

OSA = obstructive sleep apnea, TRT = total recording time, TST = total sleep time, REM = rapid eye movement, IQR = interquartile range, MAE = mean absolute error. A statistically significant difference (*p*<0.05) between manual and automatic sleep staging is denoted with an asterisk (*). A statistically significant difference (*p*<0.05) between the non-OSA group and each of the OSA groups is denoted with a dagger (†).

no OSA: AHI < 5, mild OSA: 5 ≤ AHI < 15, moderate OSA: 15 ≤ AHI < 30, severe OSA: AHI ≥ 30.

### Sleep parameters

Medians and inter-quartile ranges for TST, SE, and WASO computed using manual PSG-based and automatic PPG-based hypnograms in each OSA severity category are shown in Table 4. The medians of TST and SE computed from the automatic PPG-based hypnograms using different epoch-to-epoch intervals were in line with the medians computed from the manual PSG-based hypnograms. On the other hand, the medians of WASO in each OSA severity category were overestimated by the automatic hypnograms. In all scenarios, the mean absolute error of the automatic PPG-based sleep parameters compared to manual PSG-based sleep parameters increased when the epoch-to-epoch interval was decreased.

**Table 4.** Median (inter-quartile range) for the sleep parameters for manual and automatic sleep staging grouped by the obstructive sleep apnea (OSA) severity categories. The parameters are calculated for the test set (*n* = 175). Mean absolute error (MAE) compared to manual scoring is reported for automatic models

		TST (min)		SE (%)		WASO (min)	
		Median (IQR)	MAE	Median (IQR)	MAE	Median (IQR)	MAE
No OSA (*n* = 29)	**Manual**	330.5 (280.5–365.0)	–	73.5 (64.8–82.7)	–	80.0 (59.5–111.0)	–
	**30 s auto**	320.5 (279.0–358.0)	24.5	72.0 (61.6–78.4)	5.6	97.0 (53.5–131.5)*	26.2
	**15 s auto**	317.8 (264.0–362.2)	29.6	71.5 (60.4–79.0)	6.8	102.0 (75.2–146.0)*	32.6
	**5 s auto**	334.9 (309.4–370.8)	37.5	76.3 (69.1–81.3)	8.5	105.8 (78.4–136.5)*	41.6
Mild OSA (*n* = 42)	**Manual**	343.0 (270.0–371.1)	–	77.6 (62.3–84.4)	–	85.8 (61.1–133.4)	–
	**30 s auto**	330.8 (289.5–366.1)*	26.8	72.8 (64.8–81.4)*	6.0	94.2 (62.2–120.8)	24.0
	**15 s auto**	326.2 (280.0–350.9)*	29.5	70.5 (61.8–77.8)*	6.6	109.2 (79.4–141.9)*	27.7
	**5 s auto**	328.8 (296.5–353.1)	35.1	73.2 (67.3–76.9)	7.7	111.2 (89.5–143.0)*	33.9
Moderate OSA (*n* = 43)	**Manual**	294.5 (254.2–329.8)†	–	65.9 (58.2–76.5)	–	125.5 (77.2–170.5)†	–
	**30 s auto**	289.5 (245.0–331.0)	27.6	66.0 (55.8–76.1)	6.0	130.5 (72.5–168.0)	26.1
	**15 s auto**	285.0 (234.4–321.4)*†	34.1	64.5 (52.6–70.3)*†	7.5	137.0 (88.6–201.5)*†	33.6
	**5 s auto**	297.2 (264.1–325.5)†	38.7	66.4 (58.9–73.2)†	8.5	141.3 (115.0–184.1)*†	36.8
Severe OSA (*n* = 61)	**Manual**	287.0 (212.0–334.5)†	–	66.0 (48.2–76.1)†	–	128.0 (90.0–182.5)†	–
	**30 s auto**	296.0 (230.5–328.5)	36.3	67.1 (53.5–74.2)	8.2	122.5 (98.0–165.5)†	34.2
	**15 s auto**	277.8 (212.8–312.0)†	34.7	63.0 (45.6–69.0)†	7.8	148.5 (120.8–207.8)*†	36.2
	**5 s auto**	286.2 (246.8–322.0)†	51.1	65.5 (55.2–70.6)†	11.4	147.0 (129.6–185.8)†	47.3

TST = total sleep time, SE = sleep efficiency, WASO = wake after sleep onset, OSA = obstructive sleep apnea, IQR = interquartile range, MAE = mean absolute error. A statistically significant difference (*p* < 0.05) between manual and automatic sleep staging is denoted with an asterisk (*). A statistically significant difference (*p* < 0.05) between the non-OSA group and each of the OSA groups is denoted with a dagger (†).

no OSA: AHI < 5, mild OSA: 5 ≤ AHI < 15, moderate OSA: 15 ≤ AHI < 30, severe OSA: AHI ≥ 30.

### Assessment of sleep continuity using survival analysis

In the analysis of sleep continuity, the five-stage model was used since it provided the highest accuracy on the classification of wake (Figure 1). The test set (*n* = 175) was used for the survival analysis. In the OSA severity grouping, the clinical diagnoses based on manually scored PSGs were used. With both manually and automatically scored hypnograms, the hazard ratios (HRs) for decreased mean continuous sleep compared to the non-OSA group were larger when the OSA severity increased (Table 5). When decreasing the epoch-to-epoch interval, the differences between the HRs of different OSA severity groups increased. The HRs for PSG-based manually scored hypnograms were 1.18, 1.78, and 2.90 for mild, moderate, and severe OSA, respectively. With the PPG-based automatic scoring with 5-s epoch-to-epoch interval, the corresponding HRs were 1.70, 3.30, and 8.11.

**Table 5.** Hazard ratios for decreased mean continuous sleep in each obstructive sleep apnea (OSA) severity category versus the non-OSA group on the test set (*n* = 175) using the five-class automatic model. Results from visual inspection-based manual scoring are also provided. The *p*-values are reported for each OSA severity category compared to the non-OSA group

	OSA severity	Hazard ratio	95% CI	*p*-value
Manual	**Mild**	1.18	0.73–1.91	0.50
	**Moderate**	1.78	1.10–2.86	0.02
	**Severe**	2.90	1.85–4.54	<0.01
30 s auto	**Mild**	1.08	0.67–1.75	0.74
	**Moderate**	1.45	0.90–2.35	0.13
	**Severe**	2.75	1.74–4.35	<0.01
15 s auto	**Mild**	1.26	0.78–2.03	0.35
	**Moderate**	2.02	1.24–3.30	<0.01
	**Severe**	5.68	3.47–9.31	<0.01
5 s auto	**Mild**	1.70	1.04–2.79	0.03
	**Moderate**	3.30	1.98–5.51	<0.01
	**Severe**	8.11	4.82–13.65	<0.01

AHI = apnea-hypopnea index, OSA = obstructive sleep apnea (no OSA: AHI < 5, mild OSA: 5 ≤ AHI < 15, moderate OSA: 15≤AHI<30, severe OSA: AHI ≥ 30), CI = confidence interval

**Figure 3.**
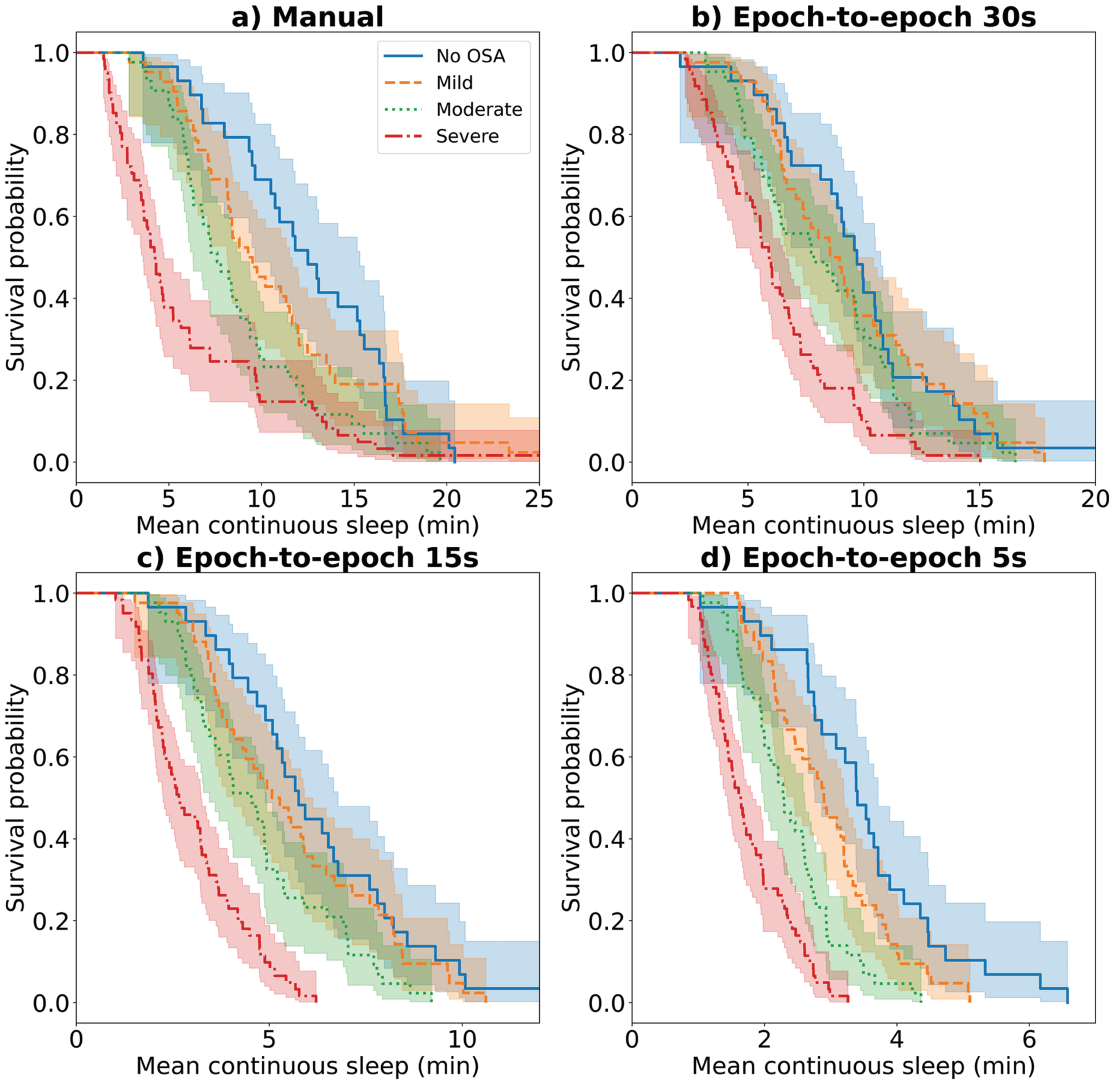
Kaplan–Meier plots illustrating the fraction of patients with more than a certain amount of mean continuous sleep in each OSA severity category. The plots are shown for (a) PSG-based manual sleep staging and PPG-based automatic sleep staging with epoch-to-epoch intervals of (b) 30, (c) 15, and (d) 5-s on the test set (*n* = 175). The colored areas around the curves denote the 95% confidence intervals.

Kaplan-Meier plots for each scenario are shown in Figure 3. With the PSG-based manually scored hypnograms, the survival curves for each OSA severity category were clearly distinct. With the automatic PPG-based model with a 30-s epoch-to-epoch interval, the mild OSA patients’ survival curve overlapped with the non-OSA curve. In contrast, with the 5-s epoch-to-epoch interval, all OSA severity categories were well separated. In addition, it is evident from the Kaplan–Meier plots that the mean continuous sleep estimated by the deep learning models decreased drastically when the epoch-to-epoch interval was decreased.

## Discussion

In the present work, OSA-related sleep fragmentation was assessed with Cox proportional hazards modeling of mean continuous sleep utilizing PPG-based automatic sleep staging. The results were compared with manual PSG-based sleep staging analyses. The hazard ratios for decreased mean continuous sleep increased along with increasing OSA severity with both automatic PPG-based and manual PSG-based analyses. This supports the first hypothesis that the automated PPG-based sleep staging models can be used to differentiate between the OSA severity categories in terms of sleep continuity. Thus, it can be reasoned that the PPG signal captures the sleep fragmentation induced by OSA-related breathing obstructions. The differences between the hazard ratios for decreased mean continuous sleep for mild, moderate, and severe OSA compared to the non-OSA group further increased when shorter epoch-to-epoch intervals were used. This is in line with the second hypothesis of the present work that a denser temporal resolution of the sleep staging would highlight the differences between the OSA severity categories with respect to sleep fragmentation.

The second aim was to improve the accuracy of automatic PPG-based sleep staging. Compared to our previous work [19], the accuracy in three-, four-, and five-stage classification on the test set increased from 64.1%, 68.5%, and 80.1% to 68.7%, 74.1%, and 83.3%, respectively. The performance of the PPG-based automated sleep staging model is remarkable, considering that the PPG signal is not utilized in the manual scoring of the sleep stages. In the five-stage classification, the accuracy of classifying REM sleep (87%) is particularly high compared to our previous PPG-based sleep staging results (69%) [19]. However, the overall performance of the PPG-based five-stage classification is still not on the level of PSG-based sleep staging, especially in the case of N1 and N3 sleep. In the case of N1 sleep, there may not be consistent hemodynamic changes compared to wakefulness and N2 sleep. It should be noted that the interrater agreement for scoring N1 sleep is particularly low also with manual EEG-based scoring [8]. Similarly, the slow wave activity of the brain, which is the main characteristic of N3 sleep, may not be reflected in the PPG signal, leading to misclassification of N3 sleep as N2 sleep. Thus, further studies are required to the application of the PPG-based models when investigating the overall sleep architecture.

The main contribution in the present work that accounts for the increased accuracy of PPG-based REM sleep classification compared to our previous work [19] was the use of a more sophisticated feature extractor CNN. With CNNs that consist of blocks of consecutive convolutional layers and occasional pooling layers, the number of parameters grows quickly to the extent that computational resources, especially the GPU memory, become a major limiting factor. In addition, when increasing the depth of the network, the gradients of layer inputs with respect to the loss tend to become smaller during back propagation. The mobile inverted residual bottleneck (MBConv) used in the EfficientNet, first introduced in the MobileNetV2 architecture [38], attempts to overcome these issues in three ways. First, instead of standard convolutions, computationally more lightweight depthwise separable convolutions are used [38]. Secondly, a linear bottleneck is used at the end of each block to reduce the number of channels passed down to the next block. Thirdly, skip connections are added from the input to the bottleneck output of the MBConv blocks for improved gradient flow. Using the MBConv blocks in the present work allowed us to significantly increase the depth of the feature extractor CNN with the same computational resources compared to our previous work [19].

According to our previous study, the REM sleep classification accuracy is comparable to EEG-based automatic sleep staging (91%), which is on par with the clinical interrater reliability [22]. Since the accuracy of identifying REM sleep is high, the PPG-based sleep staging model could be used to study REM-related phenomena, such as REM-related OSA and REM sleep fragmentation. The increasingly common home sleep apnea tests (HSAT) do not include EEG, but the PPG signal is recorded. Thus, with the prevalent methodology of EEG-based sleep staging the diagnosis of REM-related OSA cannot be done with HSATs. Therefore, it would be extremely valuable to accurately identify REM-sleep with the HSATs using only the PPG signal.

When utilizing supervised machine learning techniques, the quality of the output labels is of paramount importance. The fact that the interrater reliability of PSG-based manual scoring of sleep stages by experienced professionals is generally around 80% to 85% [5], is an issue in the supervised approach in general. In addition to the moderate interrater reliability, manual sleep staging is a very time-consuming task requiring a lot of expertise. This complicates the collection of large, high-quality datasets for supervised learning. To increase the quality of the labels and to speed up the data acquisition, the scoring rules and practices may require a revision to make the scoring process less ambiguous and easier to automate. A further step would be to develop methods for assessing sleep that do not depend on manual scoring at all. For example, unsupervised learning could be used on the PSG signals to derive sleep characteristics that capture the variance in the nocturnal PSG signals more optimally than the current visual inspection-based scoring rules. These features could correlate better with the effects of sleep deprivation such as daytime sleepiness; however, this warrants further study.

Pulse oximetry has a lot of potential for sleep analytics and diagnostics of sleep disorders as it is simple to measure and already used in various monitoring devices and applications. Since PPG measurements are easy to conduct, acquisition of larger PPG signal datasets without the manually scored PSGs is feasible. This opens possibilities to utilize semi-supervised learning with large amounts of unlabeled PPG signals and a smaller number of PPG signals with corresponding manual PSG-based labels. Thus, any dataset which includes PPG signals could be used to increase the amount of training data, regardless of whether the corresponding hypnograms are available. Semi-supervised learning has been performed with good results for example using generative adversarial networks (GANs) [39] and ladder networks [40]. In the era of consumer-grade self-tracking wearables such as smart watches, armbands, and rings, the use of deep learning-based semi-supervised methods for tasks such as sleep staging will become increasingly important.

In the prevalent sleep staging methodology, sleep is discretized to arbitrary length (usually 30-s) epochs, mainly for practical reasons to reduce the amount of work in manual scoring. In the present work, when the epoch-to-epoch interval with automatic sleep staging was artificially decreased, better differentiation was achieved between the OSA severity categories in terms of mean continuous sleep (Table 5). This finding supports the hypothesis that using the 30-s non-overlapping epochs in sleep staging does not fully capture the OSA-related sleep fragmentation. Especially with the severe OSA patients, there may be short periods of wake that are divided to two consecutive 30-s epochs such that both epochs will be scored as sleep. Using overlapping 30-s epochs with shorter epoch-to-epoch interval, those short periods of wake spanning two traditional epochs can be detected.

As seen in Figure 2, the models tend to predict increasingly fragmented sleep when the epoch-to-epoch interval is shortened. This leads to decreased mean duration of continuous sleep for all OSA severity groups as also seen in the Kaplan-Meier plots (Figure 3). However, the hazard ratios for decreased mean continuous sleep increased more rapidly with more severe OSA (Table 5). If the models would overestimate sleep fragmentation to the same extent in all OSA severity groups, as well as with the healthy subjects, the hazard ratios would not increase, since we always compare to the non-OSA group. To further investigate the overestimation of sleep fragmentation, denser-resolution manual PSG-based scorings would be needed. This underlines the need for new methods to produce higher resolution hypnograms for more detailed assessment of sleep fragmentation related to OSA.

One limitation of the present work is the amount of data. Although the main dataset B used in this study is large in the context of sleep research (*n* = 877), it is relatively small in the context of deep learning. Especially when the patients are divided into OSA severity groups and only the test set is considered, the sample sizes become small. For example, the number of patients in the non-OSA group in the test set was only 29 (Table 1). This is a limiting issue when analyzing the distributions of patient-wise variables, such as the sleep parameters or mean continuous sleep. With the epoch-based metrics, such as the overall sleep staging accuracies, this is not as problematic since on average there are hundreds of epochs for each patient.

In conclusion, the differences in hazard ratios for decreased mean continuous sleep between the OSA severity categories were increased when the epoch-to-epoch interval was decreased (Table 5). The hypnograms with higher temporal resolution were achieved by overlapping the 30-s epochs before classification with the automatic PPG-based sleep staging model. This indicates that using a shorter epoch-to-epoch interval with the automatic hypnograms better captures the OSA-related sleep fragmentation. On the other hand, decreasing the epoch-to-epoch interval increased the mean absolute error between the manual PSG-based and automatic PPG-based sleep parameters and sleep stage percentages (Table 4). Thus, although there are inconsistencies between the manual PSG-based and automatic PPG-based sleep parameters when the epoch-to-epoch interval is decreased, the increased resolution of the hypnograms better reveals the differences in sleep fragmentation between the OSA severity categories.
